# An Integrative Analysis of Transcriptome and GWAS Data to Identify Potential Candidate Genes Influencing Meat Quality Traits in Pigs

**DOI:** 10.3389/fgene.2021.748070

**Published:** 2021-10-21

**Authors:** Xianxian Liu, Junjie Zhang, Xinwei Xiong, Congying Chen, Yuyun Xing, Yanyu Duan, Shijun Xiao, Bin Yang, Junwu Ma

**Affiliations:** State Key Laboratory of Pig Genetic Improvement and Production Technology, Jiangxi Agricultural University, Nanchang, China

**Keywords:** pig, meat quality, transcriptome, QTT, eQTL, GWAS, candidate gene

## Abstract

Understanding the genetic factors behind meat quality traits is of great significance to animal breeding and production. We previously conducted a genome-wide association study (GWAS) for meat quality traits in a White Duroc × Erhualian F2 pig population using Illumina porcine 60K SNP data. Here, we further investigate the functional candidate genes and their network modules associated with meat quality traits by integrating transcriptomics and GWAS information. Quantitative trait transcript (QTT) analysis, gene expression QTL (eQTL) mapping, and weighted gene co-expression network analysis (WGCNA) were performed using the digital gene expression (DGE) data from 493 F2 pig’s muscle and liver samples. Among the quantified 20,108 liver and 23,728 muscle transcripts, 535 liver and 1,014 muscle QTTs corresponding to 416 and 721 genes, respectively, were found to be significantly (*p* < 5 × 10^−4^) correlated with 22 meat quality traits measured on longissiums dorsi muscle (LM) or semimembranosus muscle (SM). Transcripts associated with muscle glycolytic potential (GP) and pH values were enriched for genes involved in metabolic process. There were 42 QTTs (for 32 genes) shared by liver and muscle tissues, of which 10 QTTs represent GP- and/or pH-related genes, such as *JUNB*, *ATF3*, and *PPP1R3B*. Furthermore, a genome-wide eQTL mapping revealed a total of 3,054 eQTLs for all annotated transcripts in muscle (*p* < 2.08 × 10^−5^), including 1,283 *cis*-eQTLs and 1771 *trans*-eQTLs. In addition, WGCNA identified five modules relevant to glycogen metabolism pathway and highlighted the connections between variations in meat quality traits and genes involved in energy process. Integrative analysis of GWAS loci, eQTL, and QTT demonstrated *GALNT15/GALNTL2* and *HTATIP2* as strong candidate genes for drip loss and pH drop from postmortem 45 min to 24 h, respectively. Our findings provide valuable insights into the genetic basis of meat quality traits and greatly expand the number of candidate genes that may be valuable for future functional analysis and genetic improvement of meat quality.

## Introduction

Meat quality is important to both meat processing industry and consumer’s purchasing attitude, while meat quality traits are controlled by multiple genes as complex quantitative traits (Andersson and Georges, 2004). Therefore, improving pork quality is a priority and challenge task in modern pig breeding (Moeller et al., 2010; Gallardo et al., 2012; Becker et al., 2013; Nonneman et al., 2013). The genetic mechanisms that underlie meat quality traits are still largely unknown. Exploration of genes and gene networks related to meat quality traits in pigs through transcriptomic analysis will increase our knowledge of the genetic mechanisms of meat quality traits.

To date, GWAS have successfully identified a large number of genetic loci associated with meat quality traits (Ma et al., 2009; Luo et al., 2012; Nonneman et al., 2013; Liu et al., 2015; Xiong et al., 2015). They also discovered some promising candidate genes affecting target meat quality traits, such as *GLUL* for pH and *ITGB1* for cooking loss (Nonneman et al., 2013). However, many GWA studies have demonstrated that most trait-associated loci likely resulted from regulatory mutations rather than amino acid substitutions within gene, and even the genes closest to GWAS peak SNPs are frequently not causative genes underlying the studied traits (Tasan et al., 2015). The common SNPs detected by current GWAS only explain a small proportion of heritable variability and provide limited insights into biological pathways and genetic mechanisms for meat quality traits. Since genotypes give rise to organismal phenotypes through intermediate molecular phenotypes (such as gene expression), in addition to genomics, systematic analysis of multiple omics is very helpful to decipher GWAS signals.

Recently, genetic analysis of expression profiles have proved to be a popular and promising strategy for elucidating the genetic basis of complex traits (JU, 2008). Integration of expression profiles with genotyping data in segregating groups allows the mapping of expression quantitative traits loci (eQTL) which has potential to map the mutations on the level of DNA affecting the mRNA expression (Wimmers et al., 2010; Drag et al., 2019; Carmelo and Kadarmideen, 2020) and provides more biological insights into GWAS findings. Up to now, lots of studies have demonstrated that jointing GWAS and eQTL can facilitate the identification of causative genes/mutations or biological pathways (Kogelman et al., 2014; Puig-Oliveras et al., 2016) affecting studied traits. For instance, Ma et al. (2014) performed an integrative analysis of GWAS and eQTL to identify the causal variant for GP, pH24h, and drip loss of pork. Xiong et al. (2015) characterized *SDR16C5* as the important candidate gene for pig growth trait by jointing QTL mapping and gene expression analysis. Gonzalez-Prendes et al*.* (2017) observed a number of *cis*-eQTL that co-localized with QTL regions for meat quality.

To our knowledge, Ponsuksili et al. (2008), for the first time, performed quantitative trait transcript (QTT) analysis between gene expression profiles in muscle and pork drip loss in Duroc × Pietrain F2 pigs. Using White Duroc × Erhualian F2 60K SNP data, we previously identified a number of loci for meat quality traits and prioritized some interesting potential candidate genes (Duan et al., 2009; Ma et al., 2013; Ma et al., 2014). In this study, to further investigate the genetic architecture of 22 meat quality traits, herein we firstly performed QTT analysis to explore transcripts significantly associated with different meat quality attributes; then, we surveyed the over-represented GO terms of these by functional enrichment analysis. After that, through integrative analyses of GWAS, eQTL, and QTT, we detected several promising candidate genes for the meat quality traits. Finally, co-expression gene network was conducted via WGCNA package. This study provides deeper insights into the genetic determinism of meat quality traits and would benefit the final identification of causal genes underlying these traits.

## Materials and Methods

### Ethics Statement

All procedures involving animals followed the guidelines for the care and use of experimental animals approved by the State Council of the People’s Republic of China. The ethics committee of Jiangxi Agricultural University specifically approved this study.

### Animals and Phenotypic Traits

This study involved a three-generation resource population, as described elsewhere (Ren et al., 2006; Duan et al., 2009). Briefly, a total of 1912 F2 animals were obtained from nine F1 boars and 59 F1 sows in six batches, which were the progeny derived from two White Duroc sires and 17 Erhualian dams as founders. All piglets were fed with a similar diet, raised under a consistent indoor condition. Of them, 1029 F2 individuals were slaughtered for phenotype recoding at 240 ± 5 days of age in a commercial abattoir.

Twenty-two meat quality traits, including pH, EZ-drip loss and glycolytical potential (GP)-related components were measured on the LM between the 10th rib and the last rib and/or SM from the left side of the carcass. The 22 meat quality traits are as follows: pH45min, 3, 9, 15 and 24 h, pHdrop_45 min_3 h and pHdrop_45 min_24 h of LM and SM, dripEZ_24 h and dripEZ_48 h of LM and SM, glycogen, glucose-6-phosphate, lactate, and GP concentrations of LM (Table 1). The detailed measurement procedures have been described previously (Duan et al., 2009; Ma et al., 2009). In brief, the pH values were measured in LM and SM using the Delta 320 pH Meter at 45 min, 3, 9, 15, and 24 h postmortem. The pH drop between the two time points was then calculated. Each sample was measured twice and the average value of parallel measurements was used for the further analysis. Drip loss was assayed by an EZ-Drip Loss method (Rassmussen and Andersson, 1996; Otto et al., 2004). Glycogen, glucose, glucose-6-phosphate, and lactate concentrations were determined at 30 min postmortem on LM according to the procedure as described by Duan et al. (2009). Glycolytic potential (GP) was calculated using the following formula: GP = 2× (glucose + glycogen + glucose-6-phosphate) + lactate (Monin and Sellier, 1985; Maribo et al., 1999). The total amount of glucose and glycogen was defined as residual glycogen (RG) in muscle.

**TABLE 1:** Descriptive statistics of 22 meat quality traits measured on longissimus dorsi muscle (LM) and semimembranosus muscle (SM).

Trait	No	Mean ± SD^a^	CV^b^ (%)
**LM**			
pH at 45 min postmortem (pH45 min)	756	6.41 ± 0.33	5.13
pH at 3 h postmortem (pH3 h)	759	6.27 ± 0.44	7.01
pH at 9 h postmortem (pH9 h)	763	5.92 ± 0.33	5.55
pH at 15 h postmortem (pH15 h)	761	5.75 ± 0.23	4.01
pH at 24 h postmortem (pH24 h)	764	5.67 ± 0.17	3.06
pH decline from 45 min to 3 h postmortem (pHdrop_45 min_3 h)	738	0.20 ± 0.18	87.38
pH decline from 45 min to 24 h postmortem (pHdrop_45 min_24 h)	743	0.76 ± 0.31	40.54
Drip loss after 24 h storage (DripEZ_24 h), %	884	1.10 ± 0.50	44.87
Drip loss after 48 h storage (DripEZ_48 h), %	397	1.65 ± 1.01	61.08
Residual glycogen (RG), μmol/g	957	24.06 ± 14.15	58.82
Glucose-6-phosphate (6PGlucose), μmol/g	957	0.16 ± 0.35	221.90
Lactate, μmol/g	957	88.38 ± 21.72	24.57
Glycolytic potential (GP), μmol/g	957	136.80 ± 29.26	21.39
**SM**			
pH at 45 min postmortem	763	6.54 ± 0.29	4.39
pH at 3 h postmortem	657	6.36 ± 0.33	5.13
pH at 9 h postmortem	756	6.05 ± 0.27	4.47
pH at 15 h postmortem	763	5.86 ± 0.23	3.88
pH at 24 h postmortem	768	5.76 ± 0.20	3.50
pH decline from 45 min to 3 h postmortem	651	0.18 ± 0.17	91.43
pH decline from 45 min to 24 h postmortem	761	0.78 ± 0.31	39.40
Drip loss after 24 h storage, %	848	0.91 ± 0.54	58.94
Drip loss after 48 h storage, %	398	1.08 ± 0.54	49.73

a Standard deviation.

b Coefficient of variation.

### Single Nucleotide Polymorphisms Genotyping and Genome-Wide Association Study Analysis

Genomic DNA was isolated from ear tissue with a standard phenol/chloroform extraction method and then dissolved in Tris-EDTA buffer. After examining DNA quality and concentration, a total of 1,020 animals were genotyped for 62,613 SNPs on the Illumina PorcineSNP60K Beadchip according to the manufacturer’s protocol. The quality control (QC) procedures were performed by Plink v 1.07 software (Purcell et al., 2007). Briefly, the SNPs with call rate <0.9, minor allele frequency (MAF) < 0.05, and Hardy–Weinberg equilibrium *p* < 10^−5^, as well as animals with call rate <0.9 and Mendelian inheritance error rate >0.05, were excluded from the dataset. After QC, a final set of 47,956 SNPs on all F2 pigs were used for the subsequent analyses. The association between SNPs and phenotypic values were then evaluated using *polygenic* and *mmscore* function of GenABEL v1.7 as described previously (Ma et al., 2013). SNP chromosomal positions were based on the current pig genome assembly (*Sus Scrofa* Build 11.1 assembly).

### Ribo Nucleic Acid Extraction and Digital Gene Expression Quantification

Total RNA was harvested from LM muscles and livers of 493 F2 individuals using TRIzol (Invitrogen, Carlsbad, CA, United States) and further purified with RNeasy column (Qiagen, Valencia, CA, United States) following the standard manufacturer’s protocol. RNA integrity and concentration were evaluated with the Bioanalyser 2,100 (Agilent) and the NanoDrop ND-1000 Spectrophotometer (Thermo Fisher Scientific, Carlsbad, CA, United States). Expression profiles of whole genome transcripts were assayed by digital gene expression analysis (DGE), and then the DGE quantification and DGE data processing were conducted as described by Chen et al. (2013) and Zhang et al. (2017), respectively. Briefly, mRNA was isolated from total RNA using the magnetic oligo (dT) beads (Invitrogen). Double-stranded cDNA was synthesized with oligo (dT) primers and then digested by the restriction enzymes *Nla*III and *Mme*I (New England Biolabs, Ipswich, MA, United States). The digested cDNA was ligated with two Illumina specific adaptors. Polymerase chain reaction was performed to enrich the cDNA library. After purification and denaturation, the single-chain molecules of each cDNA library were loaded onto the flowcell and sequenced on a GAII sequencer (Illumina). The pig genome reference sequence and annotated transcript set were downloaded from the database of PEDE (Pig Expression Data Explorer; http://pede.dna.affrc.go.jp/) and pig unigene in the National Center for Biotechnology Information (ftp://ftp.ncbi.nih.gov/repository/UniGene/Sus_scrofa/). After removing reads of low quality in which more than half the base’s qualities were <5, we aligned qualified reads against the reference sequences using SOAP2 (Li et al., 2009), allowing up to one mismatch in 21-bp tag sequences. The number of unambiguous clean tags for each transcript was calculated and then normalized to TPM (number of tags mapped to each gene per million clean tags) as the expression level of each transcript.

### Quantitative Trait Transcript Analysis and eQTL Mapping

Firstly, gene expression profiles and phenotypic data of meat quality traits were adjusted for sex, batch, and kinship with a robust linear regression model by using the polygenic function of GenABEL in the R software (Lourenco et al., 2011). The Pearson correlation coefficient estimation between gene expression level and phenotypic values of meat quality traits was then evaluated by using the *cor* function in R environment. In order to adjust the multiple tests, a significant threshold of *p* < 0.0005 was applied, which refers to the QTT analysis in the human obesity (Naukkarinen et al., 2010) and Drosophila melanogaster (Passador-Gurgel et al., 2007). Therefore, a transcript was defined as a quantitative trait transcript (QTT) if its association coefficient reached a *p* value of <0.0005. The functional annotations of a series of genes were performed by gene ontology (GO) with DAVID Bioinformatics Resources 6.7 (https://david.ncifcrf.gov/summary.jsp). eQTL mapping was performed for transcripts in muscle using mixed linear model implemented by the *mmscore* function of GenABEL package in R software as described by Chen et al. (2013). In brief, sex and batch were considered as fixed effects, the genetic co-variances among samples were also taken into account by fitting kinship matrix derived from genotypes of whole-genome SNP markers. A Bonferroni correction was applied to adjust multiple tests. A *cis*-eQTL was defined if its peak SNP (eSNP) was located in the region from 2.5 megabase (Mb) upstream of the transcription start site to 2.5 Mb downstream of the gene end; otherwise, it was termed as a *trans*-eQTL. High linkage disequilibrium (LD) existed in the F2 special population, but LD between pairs of SNPs 2.5 Mb apart in this population was no longer significant, so 2.5 Mb was used (Xiong et al., 2015). For integrative genomic analysis, a chromosomal region around 2.5 Mb upstream and downstream of the most significant trait-associated SNPs obtained in previous GWAS analysis was defined as a QTL region. We first screened the QTTs located within QTL regions and then analyzed the associations of gene expression levels of QTTs with the SNPs within the QTL region (*cis*-eQTL).

### Weighted Gene Co-expression Network Analysis

WGCNA groups genes into modules based on patterns of co-expression, which can be linked to phenotypes by correlation analysis between trait values and the module eigengenes. All QTTs for a given trait were explored to build up a weighted gene co-expression network using the WGCNA package in R (Zhang and Horvath, 2005; Kogelman et al., 2014). In brief, all transcripts were used to construct a matrix of the Pearson correlations between gene pairs studied. The Pearson correlation matrix (PCM) then was transformed into an adjacency matrix (i.e., a matrix of connection strengths) using a power function *f*(*x*) = *x*ˆ*β*, where *x* is the absolute value of the PCM. It resulted in a “weighted” network. Analysis of scale-free topology for multiple soft thresholding powers was implemented to pick an appropriate soft-thresholding power (*β*) for network construction. In the present study, the adjacency matrix was created by calculating the Pearson’s correlation between all genes and raised to a power *β* of 2. The power *β* was chosen based on the scale-free topology criterion (Zhang and Horvath, 2005), resulting in a scale-free topology index (*R* ^2^) of 0.86. The adjacency matrix was further transformed into a topological overlap matrix to detect modules using hierarchical clustering. We then evaluated the correlations between modules and a given meat quality trait. A Bonferroni-corrected threshold of 1/(*N*_module_ × *N*_trait_), where Nmodule and Ntrait are the number of modules and meat quality traits, respectively, was adopted for the statistical significance threshold for the correlation coefficient. The network connectivity (*k*) represents the sum of a gene’s connection strengths with the other genes in the network. We defined an intramodular connectivity (*K*_in_) measure for each gene based on its correlation with the rest of genes in a given module.

## Results

### Analysis of Meat Quality Traits and Transcript Profiles in Liver and Muscle

**FIGURE 1:**
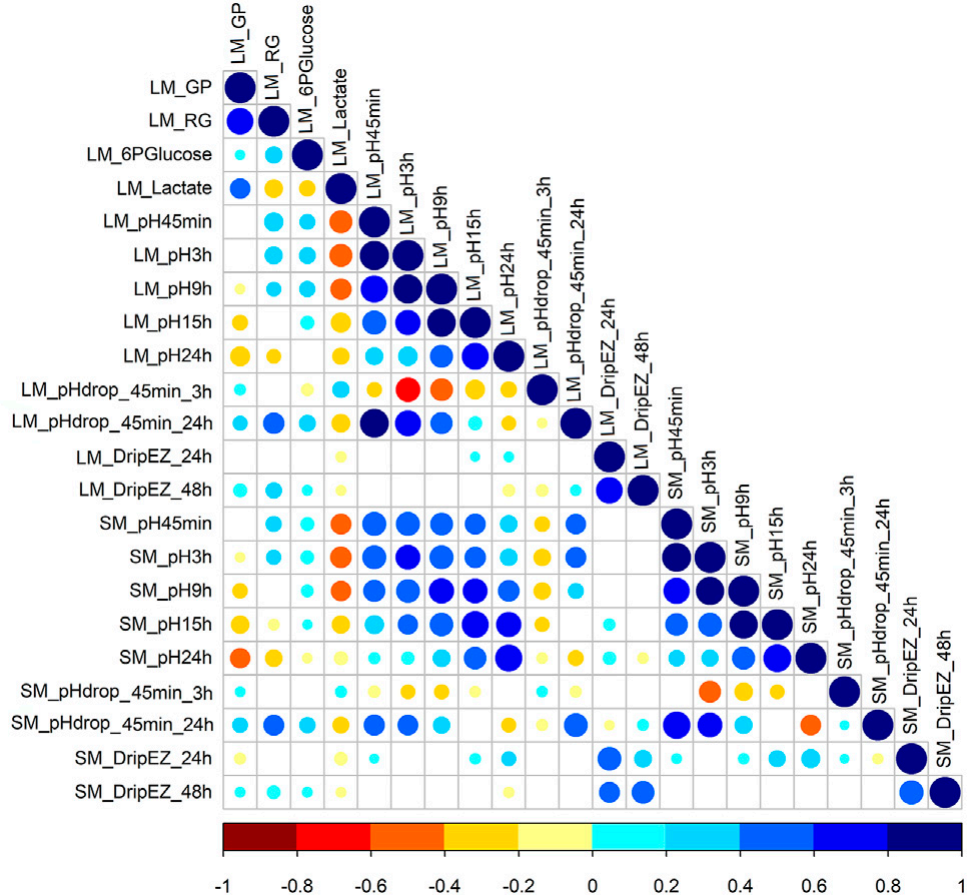
The Pearson correlation coefficients for each pair among 22 meat quality traits. The blue and red colors represent positive and negative direction of the correlation coefficient. A correlation coefficient of <−0.16 or >0.16 indicates a significant threshold of *p* < 0.05.

This study involved 22 meat quality traits recorded in the White Duroc × Erhualian F2 family, and descriptive statistics of all the studied traits are shown in Table 1. Compared with LM, SM had higher pH values measured at all the five postmortem time points (pH45 min, 3, 9, 15, and 24 h) and lower drip loss after 24 or 48 h of storage (*p* < 0.05), while the ranges of pH decline from 45 min to 3 h and from 45 min to 24 h were comparable between two muscle tissues (*p* > 0.05). We examined the correlation coefficients among the 22 meat quality traits (Figure 1 and Supplementary Tables S1, S2). The lactate content was strongly negatively (*r* < −0.5, *p* < 0.05) correlated with pH45 min and pH3h (Figure 1). In contrast, the contents of RG and 6PGlucose both were positively correlated with early post-mortem pH values while negatively correlating with pH24 h. As a result, GP showed a strong negative correlation with pH24 h (*r* = −0.39, *p* < 0.05). LM_RG was positively correlated with LM_pHdrop_45 min_24 h in a moderate degree (*r* = 0.45, *p* < 0.05). Moreover, there was a moderately high and positive correlation (*r*: 0.39–0.66) between LM and SM for the same meat quality trait.

To detect the QTTs related to meat quality traits, the expression levels of whole-genome transcripts in liver and muscle were determined by tag-based RNA sequencing. On average, about 6 million and 5 million (ranging from 3 million to 10 million reads) raw tags 35 bp in length were sequenced in liver and muscle samples, respectively. Clean reads 21 bp in length were obtained by removing the adaptors and low-quality sequences. Clean tags accounting for 93.6–98.8% (average 96.4%) of raw tags were used for the subsequent analysis. Then clean tags were mapped to the two pig transcript sequence databases: PEDE and pig unigene in NCBI. We mapped 84.2% for liver and 81.3% for muscle of clean tags to swine transcripts. After quality control, 20,108 liver and 23,728 muscle transcripts were used for further QTT analysis.

### Detection of Quantitative Trait Transcripts for Meat Quality Traits

To identify transcripts and genes that are significantly associated with meat quality traits, we evaluated the associations of the whole genome expression profiles of liver and muscle with the 22 meat quality traits liver in 493 White Duroc × Erhualian F2 pigs. Based on DGE data, 20,108 and 23,728 transcripts were qualified in liver and muscle, respectively. Among them, we identified 535 liver QTTs (corresponding to 416 genes) and 1,014 muscle QTTs (corresponding to 721 genes) that were significantly (*p* < 5 × 10^−4^) associated with those studied traits. The number of liver or muscle QTTs associated with pH, drip loss, or GP components exceeded a hundred (Table 2). Among them, 111 liver and 312 muscle transcripts were significantly associated with at least two meat quality traits.

**TABLE 2:** QTTs for pH, drip loss, and GP components identified in liver and muscle tissues.

Traits	No. of QTTs in liver	GO terms of biological process	No. of QTTs in muscle	GO terms of biological process
pH	332	Negative regulation of protein metabolic process (*p* = 4.50E-04), response to glucocorticoid (*p* = 5.30E-04), negative regulation of cellular protein metabolic process (*p* = 1.00E-03)	413	Regulation of cellular metabolic process (*p* = 1.90E-02), glycogen metabolic process (*p* = 2.60E-02)
				**KEGG_Pathway:** TNF signaling pathway (*p* = 5.60E-03)
Drip loss	141	Cellular response to fatty acid (*p* = 5.20E-04), enzyme linked receptor protein signaling pathway (*p* = 1.90E-02), cellular macromolecule metabolic process (*p* = 3.10E-02)	156	Regulation of insulin receptor signaling pathway (*p* = 1.00E-03), negative regulation of phosphorus metabolic process (*p* = 4.30E-02), regulation of cAMP-dependent protein kinase activity (*p* = 4.60E-02)
GP components	162	Lipid metabolic process (*p* = 4.30E-03), organic substance metabolic process (*p* = 4.90E-03), positive regulation of biological process (*p* = 6.20E-03)	564	Macromolecule catabolic process (*p* = 2.50E-04), macromolecule metabolic process (*p* = 1.20E-03), protein catabolic process (*p* = 3.30E-03), organic substance metabolic process (*p* = 3.40E-02), metabolic process (*p* = 3.50E-02)
				**KEGG_Pathway:** cGMP-PKG signaling pathway (*p* = 4.40E-02)

To reveal the biological function of the discovered QTT, we performed GO enrichment analysis on genes which had QTTs in liver or muscle correlated with pH, drip loss, or GP components. In muscle tissue, genes significantly associated with the GP-related traits were mainly enriched in the macromolecule catabolic process (*p* = 2.54E-04), macromolecule metabolic process (*p* = 1.20E-03), and protein catabolic process (*p* = 6.20E-03) (Table 2), and we found that genes significantly related to pH were concentrated in the GO terms of regulation of the cellular metabolic process (*p* = 1.90E-02) and glycogen metabolic process (*p* = 2.60E-02). Besides, the genes whose expression correlated with drip loss were enriched in the regulation of the insulin receptor signalling pathway (*p* = 1.00E-03), negative regulation of the phosphorus metabolic process (*p* = 4.30E-02), and regulation of cAMP-dependent protein kinase activity (*p* = 4.60E-02). The top GO biological process terms significantly overrepresenting in liver QTTs for pH, drip loss, and GP components were negative regulation of protein metabolic process (*p* = 4.50E-04), cellular response to fatty acid (*p* = 5.20E-04), and lipid metabolic process (*p* = 4.30E-03), respectively.

Forty-two QTTs were identified in both the liver and muscle tissues, representing 32 candidate genes for meat quality, 10 of which were associated with pH and/or GP (Table 3). The *JUNB* gene was significantly correlated with three meat traits including LM_pH24h, LM_GP, and SM_pH24 h. *FOS* gene was significantly associated with LM_pH3h. In addition, the most significant transcript associated with the GP in muscle was gnl. UG.Ssc.S35165160 (*p* = 1.54E-09, *r* = 0.26), and the correlation coefficient between it and the GP in liver was 0.174 (*p* = 1.54E-04). This transcript was derived from the *PPP1R3B* (Protein phosphatase 1 Regulatory Subunit 3B) gene, which encodes the catalytic subunit of the serine/threonine phosphatase-1. In human and mice, the gene-encoded protein promotes hepatic glycogen synthesis and thereby regulates fasting energy homeostasis (Dunn et al., 2006; Mehta et al., 2017; Raffield et al., 2017; Stender et al., 2017). For the 10 candidate genes, the GO-enriched terms included transcription factor activity, RNA polymerase II core promoter proximal region sequence-specific binding (*p* = 4.40E-06), skeletal muscle tissue development (*p* = 5.50E-05), and regulation of cellular macromolecule biosynthetic process (*p* = 4.20E-02) (Table 3).

**TABLE 3:** Ten candidate genes were identified to have GP- or pH-associated transcripts in both liver and muscle.

Traits	QTT	Position, bp^a^	Gene^b^	Liver tissue		Muscle tissue		GO categories
				*r* value^c^	*p* Value	*r* value	*p* Value	
LM_pH24h	ADR01_0086_A06	SSC2: 66,214,594–66,215,637	*JUNB* ^d^	0.19	9.11E-05	0.25	4.23E-07	GOTERM_MF_ALL: transcription factor activity, RNA polymerase II core promoter proximal region sequence-specific binding (*p* = 4.40E-06)
LM_GP	ADR01_0086_A06	SSC2: 66,214,594–66,215,637	*JUNB*	−0.16	4.54E-04	−0.19	5.63E-06	
SM_pH24h	ADR01_0086_A06	SSC2: 66,214,594–66,215,637	*JUNB*	0.22	8.65E-06	0.19	1.00E-04	
LM_GP	PTG01_0068_A09	SSC3: 59,176,153–59,185,280	*VAMP5*	0.16	4.24E-04	0.17	6.43E-05	
LM_pH3h	gnl.UG.Ssc.S35166542	SSC7: 98,449,508–98,453,576	*FOS*	−0.19	2.18E-04	0.22	7.67E-06	
LM_GP	gnl.UG.Ssc.S46877694	SSC9: 55,377,266–55,512,247	*ETS1*	−0.18	5.99E-05	−0.16	2.00E-04	GOTERM_BP_ALL: skeletal muscle tissue development (*p* = 5.50E-05), regulation of cellular macromolecule biosynthetic process (*p* = 4.20E-02)
SM_pH24h	gnl.UG.Ssc.S46877694	SSC9: 55,377,266–55,512,247	*ETS1*	0.18	3.96E-04	0.21	2.33E-05	
LM_RG	gnl.UG.Ssc.S42528236	SSC9: 64,031,841–64,038,694	*BTG2*	−0.20	8.35E-06	−0.16	1.29E-04	
LM_GP	gnl.UG.Ssc.S42528236	SSC9: 64,031,841–64,038,694	*BTG2*	−0.17	2.81E-04	−0.21	5.29E-07	
LM_RG	OVR01_0025_H10	SSC9: 130,772,262-130,785,599	*ATF3*	−0.25	4.47E-08	−0.18	1.95E-05	
LM_GP	OVR01_0025_H10	SSC9: 130,772,262-130,785,599	*ATF3*	−0.17	1.91E-04	−0.22	3.63E-07	
SM_pH24h	OVR01_0025_H10	SSC9: 130,772,262–130,785,599	*ATF3*	0.20	7.91E-05	0.22	1.07E-05	
LM_ pH24h	gnl.UG.Ssc.S18378970	SSC13:140,730,114-140,758,627	*TIMMDC1*	0.19	1.78E-04	0.17	4.14E-04	
LM_GP	gnl.UG.Ssc.S35165160	SSC15: 56,002,439–56,013,471	*PPP1R3B*	0.17	1.51E-04	0.26	1.54E-09	
LM_pH45min	gnl.UG.Ssc.S18549086	SSC15: 63,485,314–63,505,728	*NR4A2*	−0.18	3.04E-04	0.20	5.98E-05	
LM_pH45min	OVRM1_0062_C12	SSC16: 51,458,262–51,463,541	*DUSP1*	−0.19	2.06E-04	0.18	2.91E-04	
LM_pH3h	OVRM1_0062_C12	SSC16: 51,458,262–51,463,541	*DUSP1*	−0.22	1.33E-05	0.18	3.14E-04	

a Chromosomal location of quantitative trait transcript (QTT) according to Sus Scrofa Build 10.2 assembly.

b Annotated gene of the QTT.

c The coefficient between gene expression and phenotype value.

d The hub gene in the module 15.

### Gene Expression Network Analysis

**FIGURE 2:**
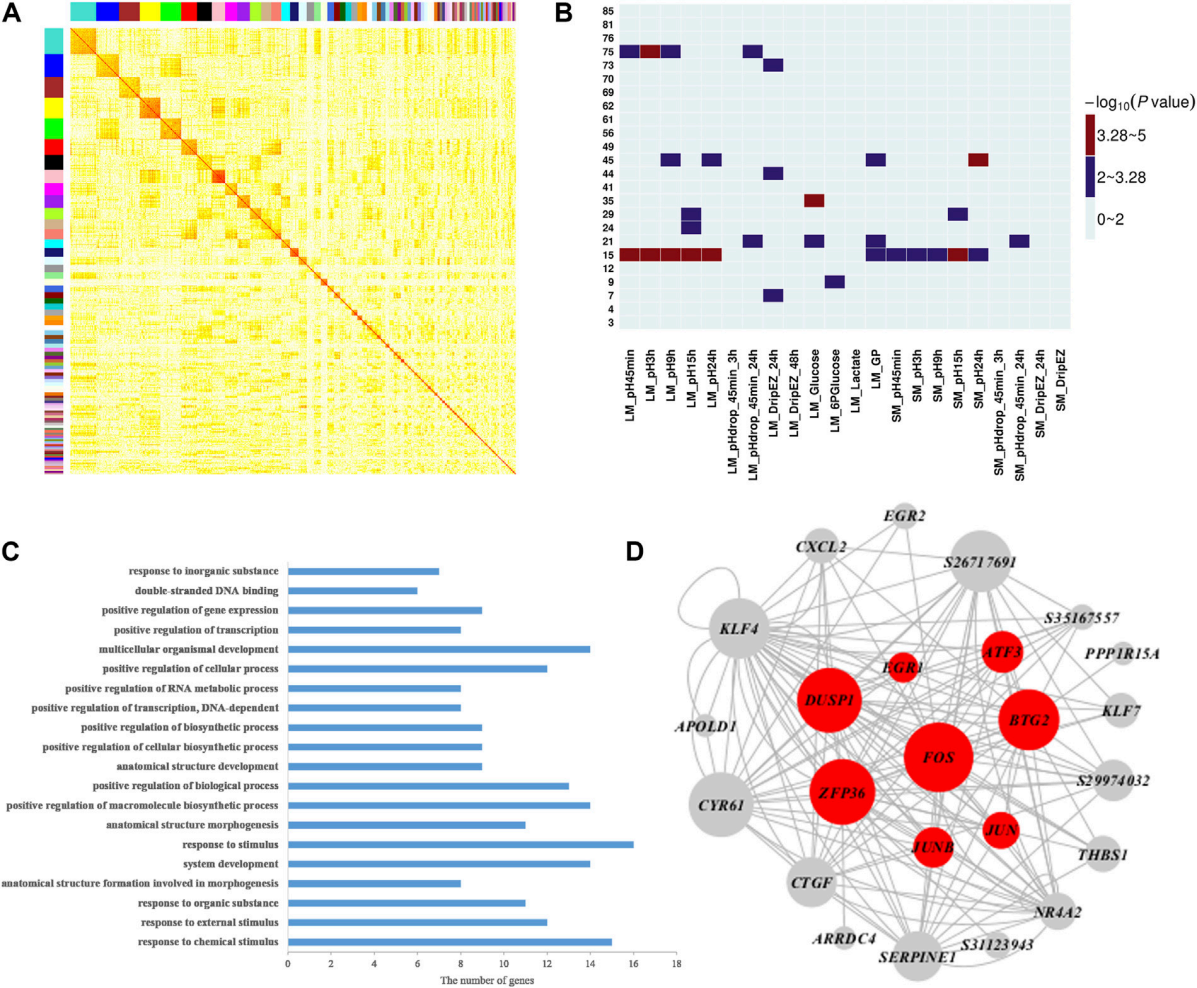
Weighted gene co-expression analysis of 17,822 transcripts in muscle. **(A)** Heat map for muscle tissue co-expression network. Each module is assigned a unique color. The modules are ordered by size, and the genes within modules in the rows and columns are sorted according to their intramodular connectivity. **(B)** Heatmap representing the significance (−log10 *p*-value) of the correlation between muscle network modules and 22 meat quality traits in muscle. **(C)** Functional annotation of module 15 genes by gene ontology (GO) analysis. The bar plot represents the gene counts within each GO category. All function or process terms listed have enrichment of corrected *p* values < 0.05. **(D)** Visualization of module 15; the top 150 connections sorted by correlation coefficients among transcripts are shown for each module. Nodes correspond to genes. When the gene symbols are unknown, transcript IDs are shown. The size of nodes represent their intramodular connectivity. Nodes in red within a module represent genes involved in glycogen metabolism pathway enriched in module 15.

To further explore the molecular mechanisms and genes related to meat quality, we constructed a weighted gene co-expression network using gene expression profiles in muscle tissue and then investigated the correlation between network modules and the 22 meat quality traits. Overall, we identified 85 muscle network modules (Figure 2A and Supplementary Table S3). Among them, four modules (modules 15, 35, 45, and 75; Figure 2B, Supplementary Tables S4, S5) were significantly correlated with at least one meat quality trait. More importantly, muscle module 15 had significant associations with 11 meat quality traits, including pH and GP values (Figure 2B). The result of GO biological enrichment analysis for the module 15 showed that the genes in this module were enriched in the categories of response to chemical stimulus (*p* = 5.20E-12), positive regulation of macromolecule biosynthetic process (*p* = 6.60E-07), and positive regulation of transcription, DNA-dependent (*p* = 1.20E-06) (Figure 2C). The co-expression network was established with the genes in module 15 (Figure 2D), and it contained the top 150 connections ranked by significance of correlation coefficients among genes in module 15. In this network, we observed that *FOS*, *JUNB*, *BTG2*, and *ATF3* belonged to hub genes, which was consistent with the findings of the QTTs for GP. Additionally, we found another four hub genes including *DUSP1*, *EGR1*, *ZEP36*, and *JUN* that were associated with GP.

### Genome-wide Detection of eQTL

**FIGURE 3:**
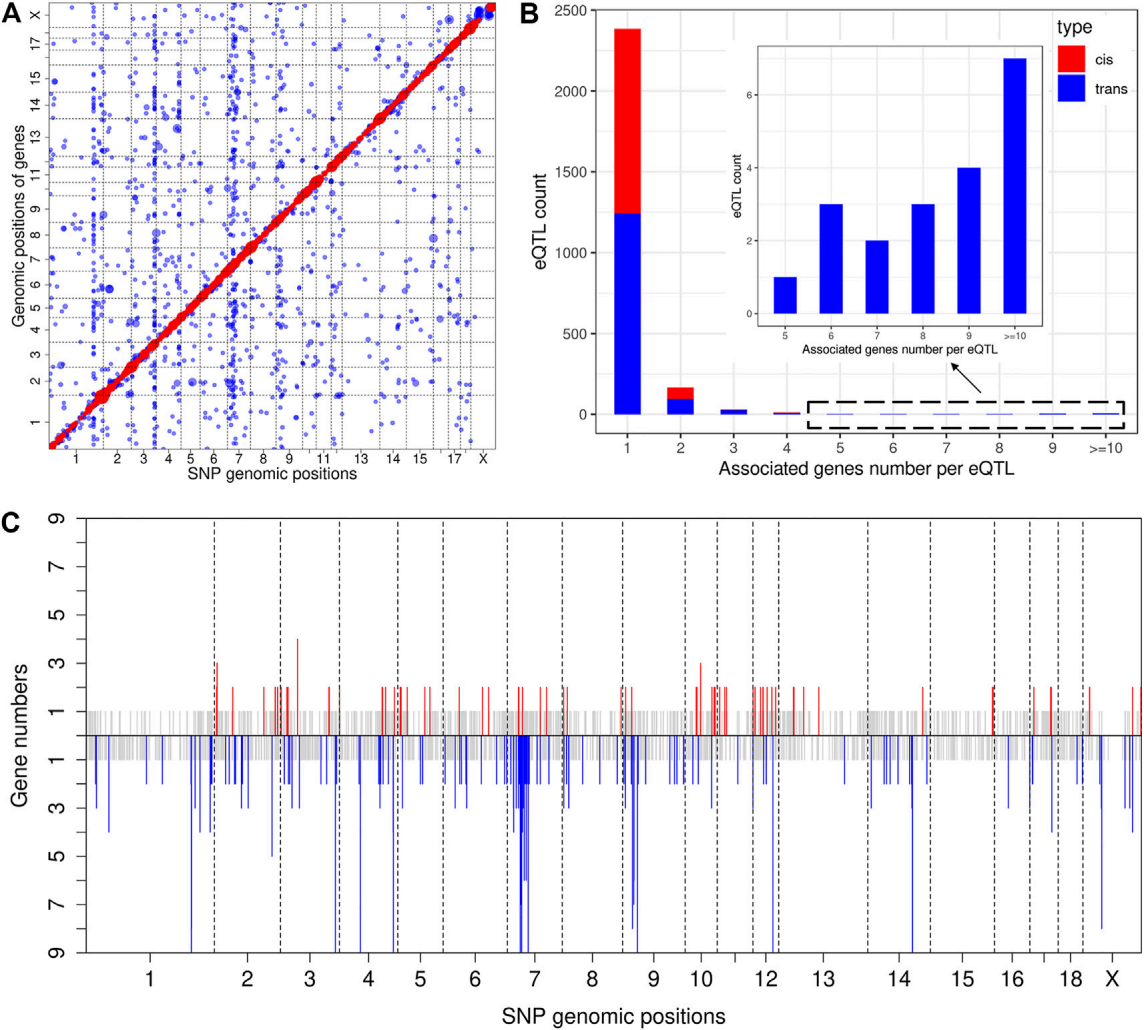
Genome-wide eQTL analysis. **(A)** Scatter plot of all eQTL. Each dot represents a SNP-gene pair, with the vertical direction linking to the SNP and the horizontal direction linking to the gene. The red and blue dots represent *cis*-eQTL and *trans*-eQTL, respectively. The size of the dot represents the degree of significance. **(B)** Analysis of eQTL pleiotropy. The X-axis of the histogram represents different groups that were classified according to the associated gene numbers per eQTL, and the Y-axis represents the eQTL count for each group. *cis*-eQTLs and *trans*-eQTLs are distinguished by red and blue color. **(C)** Distribution of eQTL hotspots. The X-axis represents the chromosome distribution of eQTLs, the Y-axis indicates the count of genes associated with each eQTL. The upper part displays the *cis*-eQTL hotspot distribution, and if the count of associated genes is 1, it is shown in grey. If greater than 2, it is shown in red. The lower part displays the *trans*-eQTL hotspot distribution, and if the count of associated genes is 1, it is shown in grey. If greater than 2, it is shown in blue.

The result of eQTL analysis for liver transcript profiling has been reported previously (Chen et al., 2013). Thus, we herein mapped eQTL using muscle transcriptome data. At a significance threshold of *p* < 2.08 × 10^−5^, we identified 3,054 eQTLs, including 1,283 *cis*-eQTLs for 1,146 genes (Supplementary Table S6) and 1771 *trans*-eQTLs for 1,569 genes (Figure 3A and Supplementary Table S7). An eQTL may regulate the expression of multiple genes, which we also refer to as pleiotropy of eQTLs (Tian et al., 2016). Descriptive statistics revealed that 73 *cis*-eQTLs (*cis*-eSNPs) and 141 *trans*-eQTLs (*trans*-eSNPs) were associated with the expression of two or more genes (Figure 3B). Of note, the eQTLs that displayed pleiotropy were distributed in some specific chromosome regions but formed clusters, which could be regarded as eQTL hotspots (Figure 3C). The most striking *cis*-eQTL with pleiotrophy was identified at 41, 817, 352 bp on SSC3 (*cis*-SNP ss131212891), which was associated with the expression of four genes (Supplementary Table S6). The *trans*-eQTLs hotspots were focused on SSC1, 3, 4, 7, 9, 12, 14, and X (Figure 3C). Strikingly, the *trans*-eSNP ss131233236 on SSC3 was found to be associated with 84 genes (Supplementary Table S7).

### Integrative Analysis of Genome-Wide Association Study, eQTL, and Quantitative Trait Transcript Data

**FIGURE 4:**
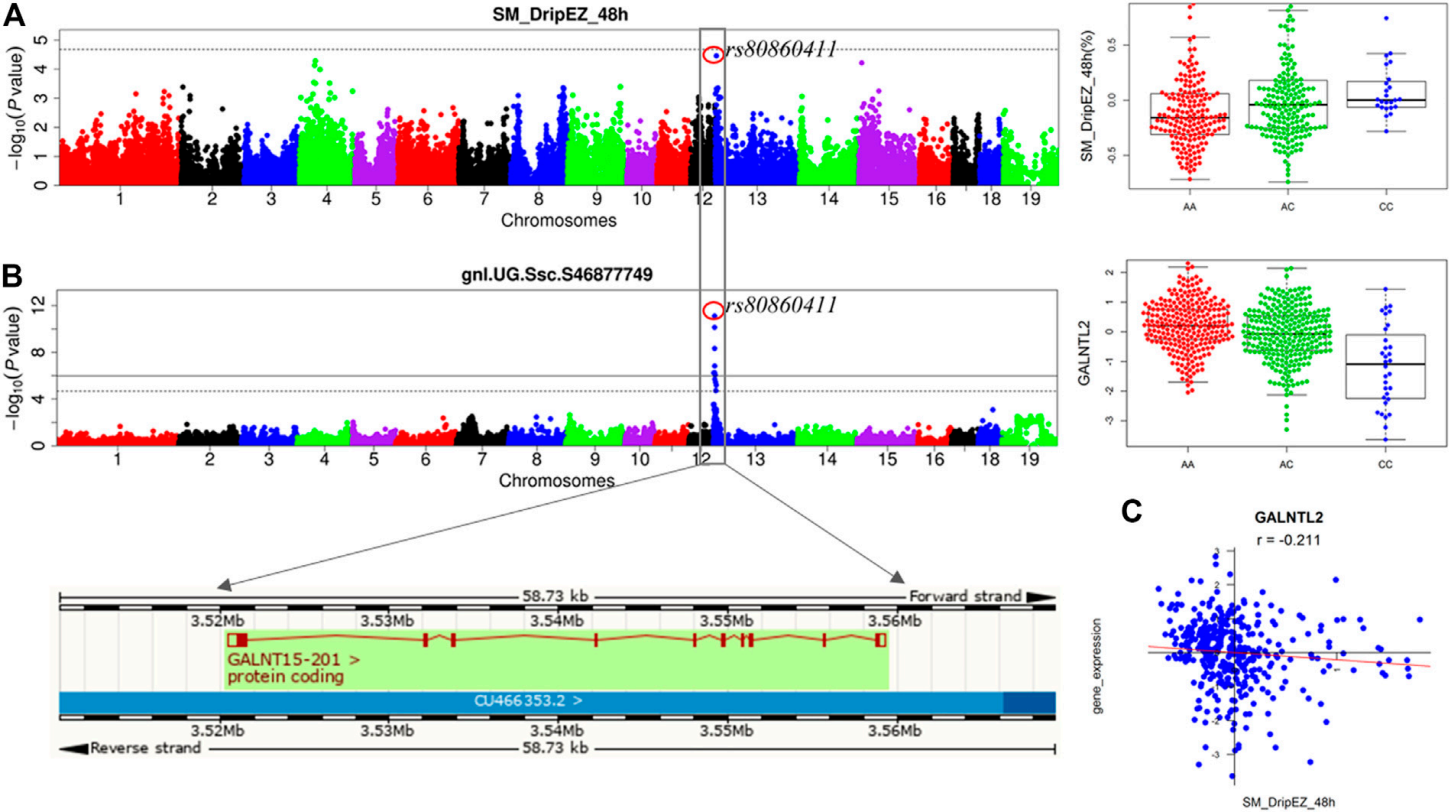
Integrating analysis of pQTL, eQTL, and QTT identify candidate gene *GALNT15* influencing drip loss. **(A)** The results for GWAS and the phenotype distribution for three genotypes of the peak SNP rs80860411 on SSC13. **(B)** The results for eQTL mapping and the gene expression level distribution for three genotypes of the peak SNP rs80860411 on SSC13. The phenotype/expression level distribution adjusting for sex and batch. **(C)** The correlation coefficient between drip loss phenotype data and gene expression level.

We previously detected the loci for the meat quality traits through linkage and GWAS analyses in the White Duroc × Erhualian F2 population (Duan et al., 2009; Ma et al., 2013). After conducting eQTL mapping using muscle transcriptome data of 493 F2 animals, we carried out an integrative analysis of GWAS, eQTL, and QTT information to identify the most likely candidate genes at the GWAS loci for meat quality. A major QTL for drip loss of SM (SM_DripEZ_48 h) was mapped on SSC13, where the most significant SNP associated with this trait was rs80860411 (*p* = 3.49E-05, Figure 4A). Interestingly, the SNP rs80860411 was just the peak *cis*-eQTL SNP for the *GALNT15* gene (*p* = 3.83E-12, Figure 4B). Moreover, the QTT analysis showed that the expression level of *GALNT15* was significant negative correlated with drip loss phenotype (*p* = 3.81E-05, *r* = −0.21; Figure 4C). Therefore, *GALNT15* was stand out as a strong candidate gene influencing drip loss.

Similarly, the GWAS signal for pHdrop_45 min_24 h was colocalized with the *cis*-eQTL for *HTATIP2*, and there was a significantly negative correlation between pHdrop_45 min_24 h phenotype and *HTATIP2* gene expression (*p* = 4.08E-04, *r* = −0.75) (Supplementary Figure S1). GO annotations related to this gene include oxidoreductase activity and NAD binding.

## Discussion

### Comparison of Trait-Related Quantitative Trait Transcripts Between Liver and Muscle

Liver and muscle are metabolically active organs and their functions have important effects on meat quality traits. Some studies have investigated the association between transcription profiles of muscle and pork quality traits (Ponsuksili et al., 2008), while few studies have identified QTTs in liver associated with pork quality traits (Zhang et al., 2017). Interestingly, the number of our observed muscle QTTs for pH, drip loss, and GP components are higher than that of liver QTTs (Table 2). A plausible explanation for this result is that changes in gene expression in muscle tissue have a more direct effect on meat quality than in liver. Unsurprisingly, the results of GO enrichment analysis on the gene sets with liver or muscle QTTs related to pH, drip loss, and GP components indicate that genes involved in the regulation of organic substance metabolism and energy metabolism in liver and muscle tissues are important for the molecular basis of variation in these meat quality traits.

Given the difference in metabolic functions between liver and muscle, comparing the QTTs and GO enrichment analysis results between liver and muscle may help in understanding the functional similarities and differences between genes associated with the same meat characteristics in the two tissues. For example, we observed 92 liver and 210 muscle QTTs associated with residual glycogen (RG) in LM. The genes in liver tissue were enriched in response to extracellular stimulus (*p* = 2.90E-06), response to starvation (*p* = 1.70E-05), cellular response to extracellular stimulus (*p* = 5.50E-05), response to nutrient levels (*p* = 9.03E-05), etc. In contrast, the genes in muscle tissue were focused on the organic substance catabolic process (*p* = 2.70E-04), muscle structure development (*p* = 6.10E-04), catabolic process (*p* = 7.20E-04), etc. The results suggest that most RG-related genes in the liver are those that can alter their expression levels in response to extracellular stimulus (such as nutrients and hormone in the blood), which in turn may influence the blood glucose level and the rate of glucose uptake in muscle, while genes in muscle that regulate catabolic process play an important role in its own RG levels. Thus, the results are consistent with the main function of the two tissues. Meanwhile, we detected 10 common QTTs for the same traits in liver and muscle tissues (Table 3). Three enriched GO terms was found for the gene set of those QTTs, including transcription activity, skeletal muscle development, and macromolecule biosynthetic process, suggesting the complexity of the genetic mechanisms of GP and pH traits.

### Comparison of Trait-Related Quantitative Trait Transcripts Between Longissiums Dorsi Muscle and Semimembranosus Muscle

Except chemical composition determined only in LM, other meat quality traits were measured in both LM and SM. On one hand, the moderate and positive correlation for the same meat quality trait between LM and SM imply that these two muscle tissues might share some common factors influencing their characteristics. Indeed, in the present study, we found that 34 liver QTTs (for 26 annotated genes) and 54 muscle QTTs (for 34 annotated genes) were associated with meat quality of both LM and SM (Supplementary Tables S8, S9). It is noted that there was only one transcript ADR01_0086_A06 in liver and muscle significantly associated with the pH24 h of LM and SM. On the other hand, although LM and SM are both characterized as fast-twitch, glycolytic skeletal muscle, there are some differences in meat quality traits between them (Table 1), which may be caused by different genetic factors (Herault et al., 2014). This notion is supported by the finding that many liver QTTs were only associated with pH24 h of LM or SM. Furthermore, the functional enrichment analysis showed that the liver genes related to pH24 h of LM were mostly enriched in small molecular metabolic process (*p* = 6.10E-04), while the liver genes for pH24 h of SM were highly concentrated at the term of the apoptotic signalling pathway (*p* = 4.40E-06). The result implied the possible potential molecular mechanism for differences in meat quality characteristics between LM and SM.

### Key Modules and Hub Genes Associated With Glycolytic Potential and pH

Our QTT and WGCNA analyses jointly identified five important candidate genes that may affect pH and/or GP, including *JUNB*, *FOS*, *ATF3*, *BTG2*, and *DUSP1* (Table 3 and Figure 2D). They were detected as the hub genes in module 15, significantly associated with pH and GP traits. Raimundo et al. (2009) showed that SRF-regulated *FOS*-*JUNB* network was downregulated in fumarate hydratase deficiency. Fumarate hydratase in the mitochondria is a Krebs cycle enzyme, which catalyzes reversible reaction: Fumaric acid + H2O ⇌ L-malate. So, *FOS*-*JUNB* pathway may play a key role in glucose and energy metabolic process. *ATF3* encodes a member of the mammalian activation transcription factor/cAMP responsive element-binding (CREB) protein of transcription factors. Several studies have demonstrated that *ATF3* modulates the immune response, atherogenesis, cell cycle, apoptosis, and glucose homeostasis (Allen-Jennings et al., 2001; Thompson et al., 2009; Kim et al., 2013; Tsai et al., 2015; Jadhav and Zhang, 2017). *BTG2* encodes a member of the BTG/Tob family, and this encoded protein is involved in the regulation of the G1/S transition of the cell cycle. *BTG2* expression was reported to play a key role in skeletal muscle growth and fat traits in pig (Feng et al., 2007; Mo et al., 2011). Knockdown of *BTG2* using lentiviral-based shRNA and siRNA severely impaired myotube formation through cell cycle arrest (Rajan et al., 2012). The protein encoded by *DUSP1* (dual-specificity phenosphatase 1) can dephosphorylate MAP kinase MAPK1/ERK2, leading to its involvement in several cellular processes. Wu et al. (2006) found that mice lacking *DUSP1* had enhanced MAP kinase activity and resistance to diet-induced obesity and exhibited unpaired insulin-mediated signalling and glucose homeostasis.

In module 15, in addition to the above-mentioned five genes, *EGR1*, *ZEP36*, and *JUN* were also identified as hub genes. EGR1 (early growth response transcription factor) functions to drive many biological processes such as differentiation, proliferation, inflammatory response, and muscle regeneration during skeletal muscle wound healing (Thiel and Cibelli, 2002; Fan et al., 2013). Recently, a number of studies have shown highly connected hub genes, like *BTG2*, *EGR1*, and *FOS*, tend to play significant roles in module organization and might be expected to play the most influential regulatory roles (Rajan et al., 2012). Raimundo et al. (2009) found that the core *FOS*-*JUNB* network have interplayed with their functional partners including *EGR1* and *ZFP36* genes, in fumarate hydratase-deficient diploient diploid fibroblasts. Ponsuksili et al. (2015) applied siRNA targeting to significantly reduce the level of a regulator gene Cxcr7 relative to control at 48 h post-transfection, then the expression of the most top hub genes (such as *EGR1*, *ZFP36*, *FOS*, *KLF4*, and *JUN*) in modules was also reduced. Therefore, in agreement with other studies (Raimundo et al., 2009; Ponsuksili et al., 2015), the hub genes and pathways in module 15 could play an important role in regulating muscle energy metabolism and meat quality traits.

### Other Identified Candidate Genes for Meat Quality Traits

Two additional candidate genes, *ETS1* and *VAMP5*, associated with GP were identified by the QTT analysis (Table 3). Like *BTG2* and *ATF3*, *ETS1* was also previously confirmed as transcriptional regulators in myogenesis (Rajan et al., 2012). The *ETS1* gene encodes a member of the ETS family of transcription factor. A study (Hua et al., 2018) demonstrated that miR-139-5p inhibits aerobic glycolysis, cell proliferation, migration, and invasion in hepatocellular carcinoma *via* a reciprocal regulatory interaction with *ETS1*. The *VAMP5* gene is a member of the vesicle-associated membrane protein (VAMP)/synaptobrevin family and the SNARE superfamily. This *VAMP* family may participate in trafficking events that are associated with myogenesis, such as myoblast fusion and/or *GLUT4* trafficking (Schwenk et al., 2010). Therefore, the *ETS1* and *VAMP5* genes could be considered as interesting candidate genes for GP.

Systematic integration of pQTL, eQTL, and QTT indicated that *GALNT15* and *HTATIP2* are key genes affecting drip loss and pHdrop_45 min_24 h (Figure 4 and Supplementary Figure S1), respectively. *GALNT15* catalyzes the initial reaction in O-linked oligosaccharide biosynthesis, the transfer of an N-acetyl-D-galactosamine residue to a serine to threonine on the protein receptor. So far, few studies have addressed this gene. A study (Abuli et al., 2011) addressed that GALNTs are interesting candidates for colorectal cancer genetic susceptibility. However, some studies of *HTATIP2* suggest that it plays a role in tumor suppress progression (Zhang et al., 2012; Yu et al., 2014). The results can help to further decipher the molecular mechanisms underlying the GWAS loci for the two traits. Moreover, the same peak SNP shared by GWAS and eQTL mapping can be applied in marker-assisted selection to achieve genetic improvement of drip loss. Apparently, our findings suggest that integrative analysis of GWAS, eQTL, and QTT is a useful and powerful way to prioritize candidate genes for meat quality traits.

## Conclusion

In conclusion, we conducted QTT analysis between genome-wide transcript profiles and 22 meat quality traits in the large scales of 493 liver and muscle samples in F2 population, respectively. Some of these QTTs have been identified as functional genes affecting meat-related traits. In addition, systems genetics study (integrating transcriptome and genomic information) revealed potential candidate genes associated with drip loss and pHdrop_45 min_24 h. Furthermore, several relevant GO terms and molecular pathway related to 22 meat quality traits were obtained. Therefore, our results shed light on the genetics of meat quality traits from a global point of view using large-scale molecular gene expression profiles, gene clusters, DE genes and QTL detection. We detected some candidate genes associated with meat quality traits and revealed some biological mechanisms related with target traits in pigs. These findings provide in-depth insights into the genetic architecture of meat quality traits, and would benefit the final identification of the underlying QTLs/genes.

## Data Availability

The raw data supporting the conclusion of this article will be made available by the authors, without undue reservation.
