# Supplementary material for: An Integrative Analysis of Transcriptome and GWAS Data to Identify Potential Candidate Genes Influencing Meat Quality Traits in Pigs
Source: Front Genet. 2021 Oct 21;12:748070. doi: 10.3389/fgene.2021.748070 (PMC8567094; doi:10.3389/fgene.2021.748070)
Supplement: Supplementary file 1 [file DataSheet1.zip › Table 8.DOCX]

**Supplementary Table 8.** 34 liver QTTs (for 26 annotated genes) were shared by LM and SM.

| **Traits** | **QTT** | **Gene** | **Position, bp** | **GO categories** |
| --- | --- | --- | --- | --- |
| **pH** |  |  |  | Amino sugar and nucleotide sugar metabolism, oxidoreductase activity, acting on the CH-CH group of donors, NAD or NADP as acceptor, Ether lipid metabolism |
| pH3h | gnl.UG.Ssc.S46877139 | *NHLRC2* | SSC14:124,162,492-124,239,727 |  |
| pH24h | gnl.UG.Ssc.S46879535 | *DECR1* | SSC4: 46,730,461-46,768,331 |  |
|  | gnl.UG.Ssc.S39995767 | *TMEM9* | SSC10: 23,583,623-23,600,097 |  |
|  | ADR01_0086_A06 | *JUNB* | SSC2: 66,214,594-66,215,637 |  |
|  | LVRM1_0052_H01 | *CHPT1* | SSC5: 82,497,437-82,544,732 |  |
|  | gnl.UG.Ssc.S35170521 | *TM7SF2* | SSC2: 7,077,018-7,082,832 |  |
|  | gnl.UG.Ssc.S35168424 | *HRG* | SSC13: 124,505,295-124,516,415 |  |
|  | gnl.UG.Ssc.S39852611 | *AGXT* | SSC15: 139,720,743-139,730,478 |  |
|  | gnl.UG.Ssc.S18383814 | *SLC35F5* | SSC15: 19,924,305-19,971,733 |  |
|  | gnl.UG.Ssc.S40115912 | *KNG1* | SSC13: 124,521,206-124,561,264 |  |
|  | LNG01_0035_G07 | *NPC2* | SSC7: 97,730,516-97,740,331 |  |
|  | OVR01_0084_A03 | *TTC23L* | SSC16: 20,443,668-20,467,619 |  |
|  | gnl.UG.Ssc.S50198146 | *UGDH* | SSC8: 30,725,289-30,759,350 |  |
|  | gnl.UG.Ssc.S35165722 | *RBBP4* | SSC6: 88,987,149-89,006,811 |  |
|  | gnl.UG.Ssc.S35165430 | *LCN2* | SSC1: 268,609,975-268,614,651 |  |
|  | gnl.UG.Ssc.S35167413 | *DBP* | SSC6: 53,986,958-53,993,020 |  |
|  | gnl.UG.Ssc.S46879272 | *SLC38A2* | SSC5: 77,113,494-77,127,714 |  |
| pHdrop_45min_24h | gnl.UG.Ssc.S35166542 | *FOS* | SSC7: 98,449,508-98,453,576 |  |
|  | gnl.UG.Ssc.S42528236 | *BTG2* | SSC9: 64,031,841-64,038,694 |  |
| **Drip loss** |  |  |  |  |
| DripEZ_24h | gnl.UG.Ssc.S35170060 | *TIMM44* | SSC2: 71,271,825-71,288,504 |  |
|  | ADR01_0061_G04 | *C9orf78* | SSC1: 269,986,279-269,995,390 |  |
|  | gnl.UG.Ssc.S39996844 | *PCF11* | SSC9: 17,297,867-17,324,710 |  |
| DripEZ_48h | gnl.UG.Ssc.S22415866 | *RENBP* | SSCX: 124,662,958-124,672,211 |  |
|  | SKNB1_0083_E10 | *BBIP1* | SSC14: 121,334,300-121,348,549 |  |
|  | gnl.UG.Ssc.S35165288 | *ERRFI1* | SSC6: 68,667,656-68,694,086 |  |
|  | DCI01_0034_H09 | *GPR1* | SSC15: 109,467,078-109,521,194 |  |
