# Supplementary material for: An Integrative Analysis of Transcriptome and GWAS Data to Identify Potential Candidate Genes Influencing Meat Quality Traits in Pigs
Source: Front Genet. 2021 Oct 21;12:748070. doi: 10.3389/fgene.2021.748070 (PMC8567094; doi:10.3389/fgene.2021.748070)
Supplement: Supplementary file 1 [file DataSheet1.zip › Table 9.DOCX]

**Supplementary Table 9.** 54 muscle QTTs (for 34 annotated genes) were shared by LM and SM.

| **Traits** | **QTT** | **Gene** | **Position, bp** | **GO categories** |
| --- | --- | --- | --- | --- |
| **pH** |  |  |  | nitrogen compound metabolic process, RNA metabolic process, regulation of biosynthetic process, ATP metabolic process, immune system process |
| pH45min | gnl.UG.Ssc.S46877694 | *ETS1* | SSC9: 55,377,266-55,512,247 |  |
|  | gnl.UG.Ssc.S31133723 | *ZFP36* | SSC6: 48,052,137-48,066,259 |  |
|  | gnl.UG.Ssc.S23691163 | *PRPF31* | SSC6: 55,991,015-56,011,441 |  |
|  | gnl.UG.Ssc.S40477283 | *NR4A2* | SSC15: 63,485,314-63,505,728 |  |
|  | gnl.UG.Ssc.S31107757 | *MT-ND6* | SSCMT: 14,739-15,266 |  |
| pH3h | gnl.UG.Ssc.S46877694 | *ETS1* | SSC9: 55,377,266-55,512,247 |  |
|  | gnl.UG.Ssc.S35168699 | *HES1* | SSC13: 131,135,530-131,140,304 |  |
|  | gnl.UG.Ssc.S31133723 | *ZFP36* | SSC6: 48,052,137-48,066,259 |  |
|  | gnl.UG.Ssc.S31107757 | *MT-ND6* | SSCMT: 14,739-15,266 |  |
|  | gnl.UG.Ssc.S39861498 | *SLC5A6* | SSC3: 111,919,312-111,976,091 |  |
|  | gnl.UG.Ssc.S40477283 | *NR4A2* | SSC15: 63,485,314-63,505,728 |  |
| pH9h | gnl.UG.Ssc.S46877694 | *ETS1* | SSC9: 55,377,266-55,512,247 |  |
|  | gnl.UG.Ssc.S40477283 | *NR4A2* | SSC15: 63,485,314-63,505,728 |  |
|  | gnl.UG.Ssc.S35168699 | *HES1* | SSC13: 131,135,530-131,140,304 |  |
|  | gnl.UG.Ssc.S40015666 | *PIM1* | SSC7: 32,788,005-32,793,881 |  |
|  | gnl.UG.Ssc.S46879631 | *ADAMTS4* | SSC4: 89,260,459-89,269,244 |  |
|  | gnl.UG.Ssc.S35334793 | *MAFF* | SSC5: 9,683,657-9,695,934 |  |
|  | gnl.UG.Ssc.S35168574 | *ALDOA* | SSC3: 18,234,775-18,263,324 |  |
| pH15h | gnl.UG.Ssc.S35334793 | *MAFF* | SSC5: 9,683,657-9,695,934 |  |
|  | gnl.UG.Ssc.S31100801 | *CPPED1* | SSC3: 30,362,596-30,486,175 |  |
|  | gnl.UG.Ssc.S46879631 | *ADAMTS4* | SSC4: 89,260,459-89,269,244 |  |
|  | gnl.UG.Ssc.S35169654 | *JMJD6* | SSC12: 4,809,118-4,814,916 |  |
|  | gnl.UG.Ssc.S35168574 | *ALDOA* | SSC3: 18,234,775-18,263,324 |  |
| pH24h | gnl.UG.Ssc.S35334793 | *MAFF* | SSC5: 9,683,657-9,695,934 |  |
|  | gnl.UG.Ssc.S35167690 | *CYP4F3* | SSC2: 61,864,575-61,884,678 |  |
|  | gnl.UG.Ssc.S35166366 | *ADM* | SSC2: 49,228,610-49,231,446 |  |
|  | gnl.UG.Ssc.S35172997 | *PRKAG2* | SSC18: 5,475,410-5,746,613 |  |
|  | LVRM1_0118_C09 | *LONRF3* | SSCX: 97,493,660-97,548,349 |  |
|  | gnl.UG.Ssc.S31100801 | *CPPED1* | SSC3: 30,362,596-30,486,175 |  |
|  | gnl.UG.Ssc.S46879631 | *ADAMTS4* | SSC4: 89,260,459-89,269,244 |  |
|  | gnl.UG.Ssc.S20116349 | *NR4A3* | SSC1: 241,569,830-241,613,629 |  |
|  | ADR01_0086_A06 | *JUNB* | SSC2: 66,214,594-66,215,637 |  |
|  | AMP01_0070_H03 | *TIPARP* | SSC13: 96,496,405-96,525,731 |  |
|  | OVRM1_0062_C12 | *DUSP1* | SSC16: 51,458,262-51,463,541 |  |
|  | gnl.UG.Ssc.S35169654 | *JMJD6* | SSC12: 4,809,118-4,814,916 |  |
|  | gnl.UG.Ssc.S18553815 | *FAM134B* | SSC16: 5,726,494-5,879,466 |  |
|  | THY01_0202_A09 | *SLC15A4* | SSC14: 26,122,356-26,154,781 |  |
|  | OVRM1_0085_E11 | *MUM1L1* | SSCX: 86,788,430-86,814,622 |  |
|  | gnl.UG.Ssc.S50239385 | *XIRP1* | SSC13: 23,867,476-23,876,850 |  |
|  | gnl.UG.Ssc.S19546121 | *CPEB2* | SSC8: 10,401,296-10,472,137 |  |
|  | gnl.UG.Ssc.S47005444 | *ABRA* | SSC4: 30,711,963-30,722,936 |  |
|  | gnl.UG.Ssc.S46916717 | *KLF4* | SSC1: 248,604,822-248,609,718 |  |
| pHdrop_45min_24h | gnl.UG.Ssc.S35170719 | *ZCCHC6* | SSC10: 28,995,574-29,061,177 |  |
| **Drip loss** |  |  |  |  |
| DripEZ_24h | LVR01_0042_A05 | *FAM193B* | SSC2: 80,395,932-80,430,071 |  |
|  | gnl.UG.Ssc.S40519776 | *IKBIP* | SSC5: 85,258,443-85,278,232 |  |
|  | gnl.UG.Ssc.S35164920 | *SUOX* | SSC5: 21,395,266-21,399,736 |  |
| DripEZ_48h | gnl.UG.Ssc.S46877749 | *GALNTL2* | SSC13: 3,330,023-3,373,481 |  |
|  | gnl.UG.Ssc.S35170907 | *MLX* | SSC12: 20,249,761-20,257,482 |  |
