# Supplementary figures and images for: An Integrative Analysis of Transcriptome and GWAS Data to Identify Potential Candidate Genes Influencing Meat Quality Traits in Pigs
Source: Front Genet. 2021 Oct 21;12:748070. doi: 10.3389/fgene.2021.748070 (PMC8567094; doi:10.3389/fgene.2021.748070)

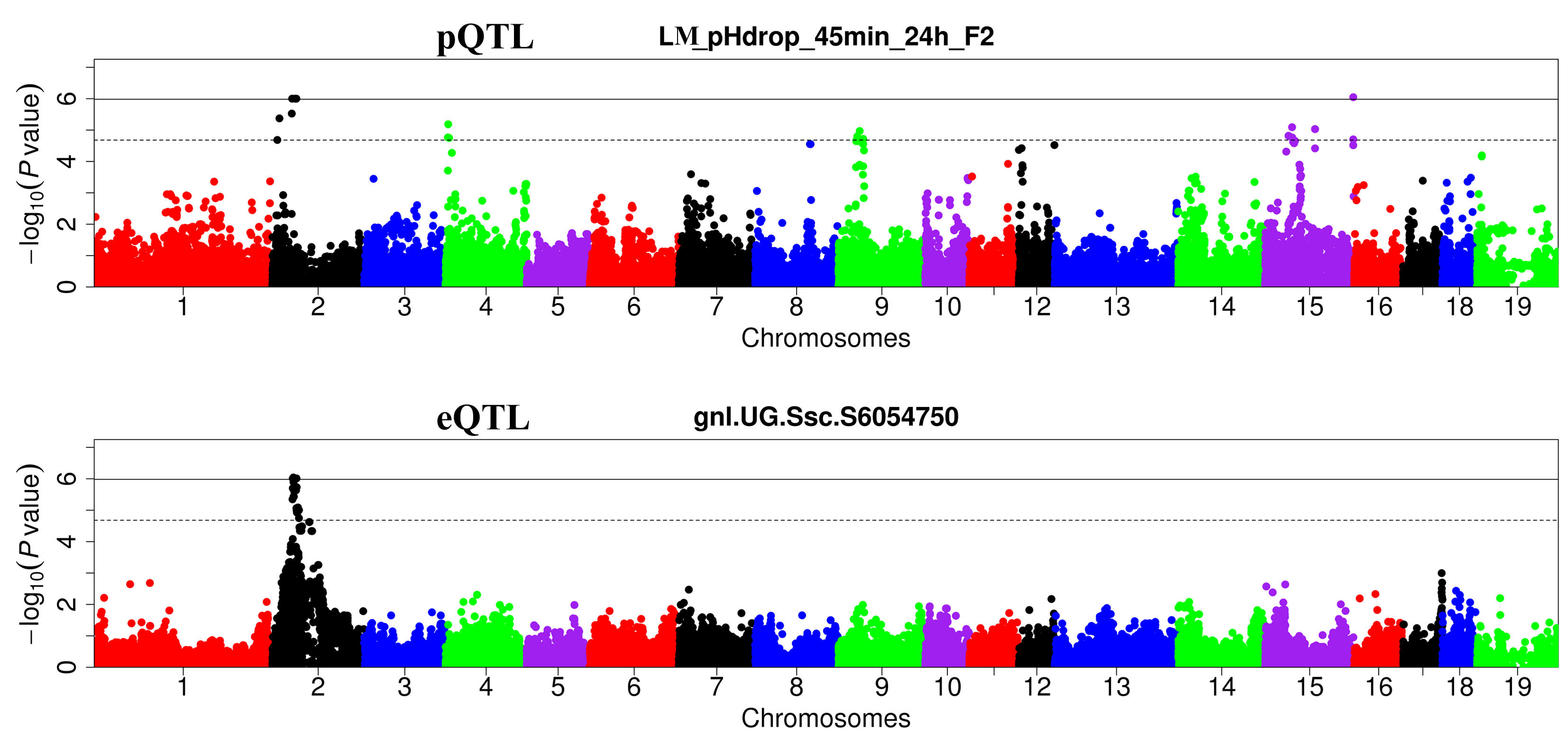

Supplement: Supplementary file 2 [file Image1.TIF]
